# Expression characterization and functional implication of the collagen-modifying Leprecan proteins in mouse gonadal tissue and mature sperm

**DOI:** 10.3934/genet.2018.1.24

**Published:** 2018-02-07

**Authors:** Sarah M. Zimmerman, Roberta Besio, Melissa E. Heard-Lipsmeyer, Milena Dimori, Patrizio Castagnola, Frances L. Swain, Dana Gaddy, Alan B. Diekman, Roy Morello

**Affiliations:** 1 Department of Physiology & Biophysics, University of Arkansas for Medical Sciences, Little Rock, AR, USA; 2 IRCSS AOU-San Martino-IST, Genoa, Italy; 3 Department of Orthopaedic Surgery, Center for Orthopaedic Research, University of Arkansas for Medical Sciences, Little Rock, AR, USA; 4 Department of Biochemistry, University of Arkansas for Medical Sciences, Little Rock, AR, USA; 5 Division of Genetics, University of Arkansas for Medical Sciences, Little Rock, AR, USA

**Keywords:** Leprecans, CRTAP, SC65, testis, ovary, sperm morphology

## Abstract

The Leprecan protein family which includes the prolyl 3-hydroxylase enzymes (P3H1, P3H2, and P3H3), the closely related cartilage-associated protein (CRTAP), and SC65 (Synaptonemal complex 65, aka P3H4, LEPREL4), is involved in the post-translational modification of fibrillar collagens. Mutations in *CRTAP*, *P3H1* and *P3H2* cause human genetic diseases. We recently showed that SC65 forms a stable complex in the endoplasmic reticulum with P3H3 and lysyl hydroxylase 1 and that loss of this complex leads to defective collagen lysyl hydroxylation and causes low bone mass and skin fragility. Interestingly, SC65 was initially described as a synaptonemal complex-associated protein, suggesting a potential additional role in germline cells. In the present study, we describe the expression of SC65, CRTAP and other Leprecan proteins in postnatal mouse reproductive organs. We detect SC65 expression in peritubular cells of testis up to 4 weeks of age but not in cells within seminiferous tubules, while its expression is maintained in ovarian follicles until adulthood. Similar to bone and skin, SC65 and P3H3 are also tightly co-expressed in testis and ovary. Moreover, we show that CRTAP, a protein normally involved in collagen prolyl 3-hydroxylation, is highly expressed in follicles and stroma of the ovary and in testes interstitial cells at 4 weeks of age, germline cells and mature sperm. Importantly, *CrtapKO* mice have a mild but significant increase in morphologically abnormal mature sperm (17% increase compared to WT). These data suggest a role for the Leprecans in the post-translational modification of collagens expressed in the stroma of the reproductive organs. While we could not confirm that SC65 is part of the synaptonemal complex, the expression of CRTAP in the seminiferous tubules and in mature sperm suggest a role in the testis germ cell lineage and sperm morphogenesis.

## 1. Introduction

The Leprecan family of proteins is composed of five members that mostly localize to the endoplasmic reticulum (ER). Three of them are prolyl 3-hydroxylase enzymes (P3H1 or Leprecan, P3H2, and P3H3) that have a highly conserved 2-oxoglutarate, ascorbate- and Fe(II)-dependent dioxygenase domain and can hydroxylate specific proline residues in the triple helical domain of fibrillar collagens. The other two members, CRTAP (Cartilage-associated protein) and SC65 (Synaptonemal complex 65 or Leprel4), lack the enzymatic domain but show significant similarity to the N-terminal portion of the P3Hs, including the presence of tetratricopeptide (TPR)-like repeats. CRTAP and P3H1 form a stable complex in the ER that also includes a third component, cyclophilin B (CYPB), a prolyl cis-trans isomerase [1]. This trimeric complex, known as the prolyl 3-hydroxylation complex, is essential for proper collagen modification and folding in the ER and ultimately for healthy bone formation. Mutations in any one of the genes coding for CRTAP, P3H1 or CYPB cause a severe skeletal dysplasia in mice and humans that is classified as recessive osteogenesis imperfecta (rOI) type VII, VIII or IX, respectively [1]–[3]. Mutations in the gene coding for P3H2 instead have been associated with recessive severe myopia in at least two distinct families and likely impact collagen modifications in the eye [4],[5]. Therefore, Leprecan genes and mutations thereof are relevant in human disease and the underlying cause appears to be primarily related to collagen alterations.

Until recently, the function of SC65 was the least understood and quite controversial findings were published on the subject. Old literature described SC65 as a protein associated with the synaptonemal complex (SC) [6], the essential structure that mediates pairing of the homologous chromosomes during the first meiotic division [7]. However, subsequent studies have assigned SC65 alternate, diverse roles including an autoantigen associated with prostate cancer, an adaptor protein for myelin protein zero (P0), a gene up-regulated in the hippocampus during sleep, and a possible interactor of PTEN [8]–[11]. Our work instead has recently shown that SC65 forms a stable complex in the ER with P3H3 and can also interact with lysyl hydroxylase 1 (LH1) and CYPB. *Sc65* is expressed at high levels in bone, cartilage and skin and at lower levels in a few other tissues including kidney and brain, and two distinct mouse models of *Sc65* loss of function consistently showed osteopenia and skin fragility [12],[13]. Lack of SC65 in osteoblasts or fibroblasts causes dramatic reduction of LH1 and P3H3 protein levels and severely reduced LH1 activity as detected by loss of collagen triple-helical lysine hydroxylation with consequent defective collagen cross-links and altered collagen fibrillogenesis in the extracellular matrix (ECM).

While SC65 was initially associated with the synaptonemal complex, no further studies confirmed this and the expression of other Leprecans in gonadal tissue has remained unexplored. In this study we quantitatively analyzed the expression of Leprecan mRNAs and proteins in both murine testis and ovary and examined SC65 and CRTAP localization at the cellular level. We confirmed in gonads the temporal co-expression of SC65 and P3H3 and of CRTAP and P3H1, which we previously described in bone and skin. Importantly, we also described the unexpected expression of CRTAP in testis germline cells and mature sperm and identify mild but significant sperm morphological defects in *CrtapKO* mice.

## 2. Materials & methods

### 2.1. Mice and ethics statement

Both *CrtapKO* and *Sc65KO* mice were generated previously [1],[13]. *CrtapKO* mice are maintained in a mixed C57BL6/J;129/SvEv genetic background, while *Sc65KO* mice are in a pure B6 background. Wild-type mice used in comparative studies were often derived from the same litter, and always within the same genetic background. Mice were housed in a pathogen-free facility, with unlimited access to water and standard rodent chow and with a 12-hour light/dark cycle. *P3h3KO* mouse tissues were generously provided by Dr. Brendan Lee, Baylor College of Medicine, Houston, TX [14].

All animal work performed in this study was conducted in accordance to local, State and US Federal regulations. The UAMS IACUC has approved the animal protocol (AUP#3349 entitled “Role of the Leprecan Genes in Skeletal Formation”) describing all the procedures performed in this study. Mice were euthanized to harvest relevant tissues according to the recommendations of the Guide for Care and Use of Laboratory Animals 8^th^ Edition.

### 2.2. Mature sperm extraction

Mature sperm samples were prepared from adult male mice (> 8 weeks old). After euthanasia, the caudal epididymides were removed. After making several cuts in the tissue with a scissors, the tissue was placed in warm, sterile PBS for 10–15 minutes to allow sperm swim-out. To fix the sperm, paraformaldehyde was added to a final concentration of 4% and incubated for 30 minutes. For trypan blue staining, equal volumes of solution containing fixed sperm and trypan blue stain 0.4% (BioWhittaker cat#17-942E) were mixed and incubated for 10 minutes. The fixed sperm, stained and unstained, were spotted onto slides and allowed to dry at room temperature, after which the slides were stored in a dark box at 4 °C. For sperm morphology assessment, trypan blue stained mature sperm from 3 WT or heterozygous and 3 *CrtapKO* male 5 month old littermates were imaged at 40x on a Nikon Eclipse E400 microscope with Olympus DP73 camera and cellSens Entry software. The numbers of abnormal and total sperm were counted by three independent, blinded researchers using ImageJ software. The proportion of sperm with abnormal morphology was compared between genotypes, and the total sperm counted was 659 for WT/Het and 618 for *CrtapKO*.

### 2.3. Real-time PCR

Total RNA was extracted from WT mouse testes of various ages and from 1 month old WT mouse cortical bone shafts from femur and tibia (after elimination of epiphyses and bone marrow). RNA extraction was performed using TriPure Isolation Reagent (Roche REF11667157001), and synthesis of cDNA was performed using the Transcriptor First Strand cDNA Synthesis Kit (Roche REF04379012001), according to the manufacturer's protocols. Absolute quantification of *P3h1*, *P3h2*, *P3h3*, *Crtap*, and *Sc65* mRNA was performed by real-time quantitative PCR using the LightCycler 1.5 (Roche), running LightCycler Software 5.0, and using the LightCycler FastStart DNA Master-PLUS SYBR Green I kit (Roche REF 03515885001) following the manufacturer's instructions. A standard curve was generated for each gene using serial dilutions of known concentrations of plasmid containing a full-length construct of the coding region of the gene. The copy number calculated for the unknowns was normalized to the total RNA. The primers and annealing temperatures used for real-time PCR are listed in Table 1 and each primer set had an amplification efficiency > 90%. The specificity of amplification products was confirmed by a single band present when the PCR products were separated on a 2% agarose gel, and a single dissociation peak on the thermal melting curve.

**Table 1.** qPCR primer sequences.

Primer	Sequence (5′ to 3′)
P3h1 Forward	ACTACAGCGCCATCCTTTACC
P3h1 Reverse	CTGGTGACAGCCTTCACTCC
P3h2 Forward	TAAAACCGTGACTGCCTCTATAA
P3h2 Reverse	TAAAGAGGGTCCAGTGTAAACCAT
P3h3 Forward	CCCCCAGCCTACACCTATCGAGACTAT
P3h3 Reverse	AGAACAGGTCGCCTCCCTTGAAGTCATC
Crtap Forward	CCCGAAGCAGTTCAGTTCTTTA
Crtap Reverse	CCAACAAGTCGTCCACATACTC
Sc65 Forward	ATGCAGCAGAACCTGGTATATT
Sc65 Reverse	GTCTGGTTGTGGTAGAGCATAG

### 2.4. Western blot

Tissues were lysed into RIPA buffer (50mM Tris-HCl pH 7.5, 150mM NaCl, 0.1% SDS, 1 mM EDTA, 0.5% sodium deoxycholate, and 1% Triton X-100) containing a cocktail of protease inhibitors including EDTA (cAMRESCO). Lysates were centrifuged (15,000 g) and supernatants collected and quantified using the Bio-Rad protein assay dye reagent (cat.# 5000006 Bio-Rad). Proteins were separated by 10% SDS-PAGE gels according to standard techniques (loading 20 µg protein for CRTAP blots and 50 µg for all others), transferred to a nitrocellulose membrane and blocked for 30 min in 5% milk in TBS-T. Primary antibodies were anti-SC65 (ProteinTech cat# 15288-1-AP, 1:500), anti-CRTAP (1:1000 [1]), anti-LEPRE1 (Novus Biologicals cat.# H00064175-M01, 1:250), anti-β-actin (Cell Signaling cat.# 3700, 1:20,000), and anti-P3H3 (ProteinTech cat.# 16023-1-AP, 1:500) diluted in 5% milk in TBS-T (also see Table S1). Membranes were incubated with primary antibody solution overnight at 4 °C, followed by washing in TBS-T. Secondary antibodies were goat anti-rabbit or anti--mouse IRDye 680LT (LICOR Biosciences cat.# 925-32210 and 922-32211, 1:20,000), and were incubated with the membranes for 1 hour at room temperature. Membranes were scanned and densitometry performed using a LICOR Odyssey instrument with Image Studio ver.5.2 software.

### 2.5. Histology, immunohistochemistry and immunofluorescence

Testes and ovaries were harvested and fixed in 10% buffered formalin, dehydrated, cleared and embedded in paraffin according to standard procedures. Paraffin embedded sections were cut at 5 microns on a Leitz1512 microtome. Sections were mounted on Silane + slides (Newcomer's Supply) using Haupt's media to allow the sections to adhere better to the slides. Immunohistochemistry staining was performed according to standard procedures, using the Vectastain Elite ABC Kit with Rabbit IgG (Vector Labs PK-6101) and Peroxidase Substrate Kit (Vector Labs SK-4100) and following the manufacturer's protocols. For antigen retrieval, slides were placed in 10 mM citrate buffer pH 6.0, heated to boiling in a microwave oven, and then allowed to cool for 30 minutes at room temperature. Endogenous peroxidase was blocked by incubating the slides in 3% hydrogen peroxide for 30 minutes. Slides were incubated overnight at 4 °C in a humid chamber with the primary antibody diluted with blocking buffer: anti-SC65 (1:500) or anti-CRTAP (1:1000). The sections were counterstained with Mayer's Hematoxylin. For IF on mature sperm, the slides were removed from 4 °C, allowed to reach room temperature, and then washed briefly in PBS. Slides were blocked in normal serum blocking buffer (3% normal goat serum, 1 mg/ml BSA, 0.1% Triton in PBS) for 30 minutes, then incubated with primary antibody overnight at 4 °C in a humid chamber for anti-SC65 (diluted 1:200) or 1.5 hours at 4 °C for anti-CRTAP (1:200). After washing, sections were incubated with secondary antibody AlexaFluor 488 goat anti-rabbit (Thermo Scientific cat.# A-11008, 1:600) for 1 hour followed by washing and mounting in DAPI Fluoromount (Southern Biotech cat.# 0100-20). Images were acquired using a Nikon Eclipse E400 microscope with Olympus DP73 camera and cellSens Entry software. For higher magnification, images were acquired on a Zeiss AxioImager microscope equipped with Zeiss AxioCam MRc5 digital camera and Zeiss Axiovision software.

### 2.6. Statistical analysis

All parameters measured are presented as mean ± standard deviation and were analyzed with the Student's t-test (using a two-tailed distribution and two-sample equal variance) or one-way ANOVA followed with Tukey's post-hoc test as appropriate. Calculations were performed utilizing Microsoft Excel with the Real Statistics add-in (www.real-statistics.com) or in SigmaPlot v.12.3. P values < 0.05 were considered statistically significant and reported as such.

## 3. Results

### 3.1. Quantification of expression of Leprecans in testis

**Figure 1.**
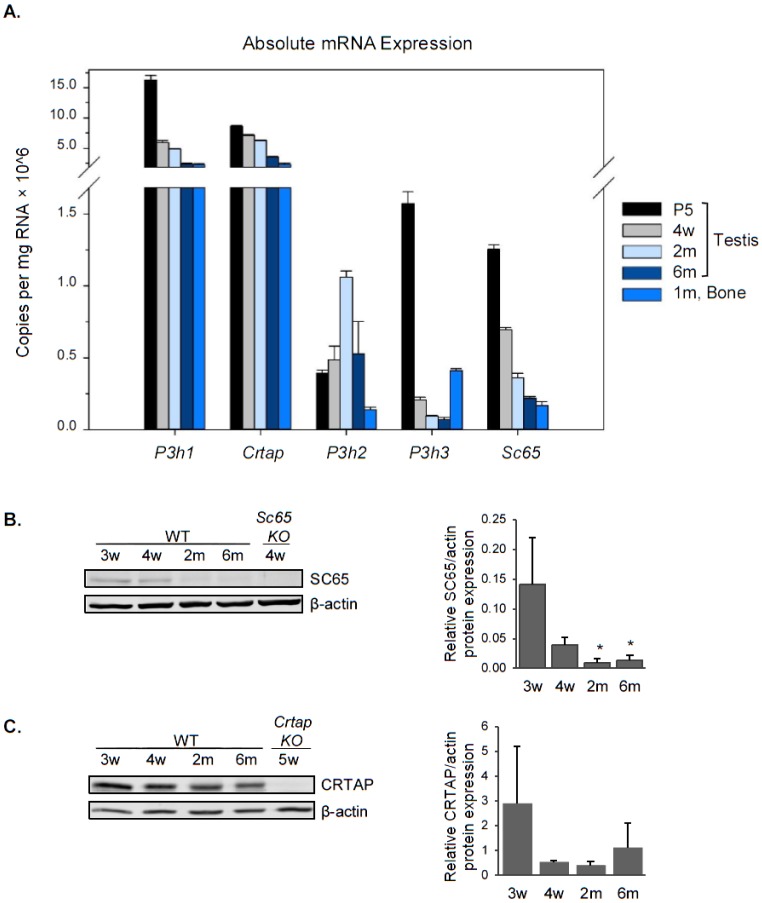
Leprecans expression in mouse testis. A. Absolute quantification of the transcript copy number normalized to total RNA for each of the five Leprecan genes in WT mouse testis at postnatal day 5 (P5), 4 weeks old (4w), 2 months old (2m) and 6 months old (6m), and in WT 1 month-old mouse cortical bone (1m, Bone). N = 1, each measurement was performed in triplicate. B. Western blot analysis for SC65 in mouse testis from WT and *Sc65KO* mice at the ages indicated. Quantification by densitometry is shown to the right, normalized to beta-actin, n = 3 replicates, * p < 0.05 compared to 3 weeks old. C. Western blot analysis for CRTAP in mouse testis from WT and *CrtapKO* mice at the ages indicated. Quantification by densitometry is shown to the right, normalized to beta-actin, n = 3 replicates.

Using real-time PCR, we first quantified the absolute mRNA expression of *P3h1, P3h2, P3h3, Crtap* and *Sc65* in mouse testis and compared it to that of cortical bone as a positive control (Figure 1A). *P3h1* and *Crtap* were the most highly expressed of the five genes in testis at every age and their expression was even higher compared to that in cortical bone. *P3h3* and *Sc65* were expressed at much lower levels in testis compared to *P3h1* and *Crtap*, and overall all of them were more expressed at postnatal day 5 (P5) compared to later age groups. *P3h2*, unlike the others, has relatively the same expression level in every age analyzed. Protein expression in testis was confirmed for SC65, CRTAP, and P3H3 by western blot and correlated well with mRNA findings (Figures 1B,C and 6A). We found that SC65 protein was present in young, 3 and 4 week-old testes, but was dramatically down-regulated starting at 2 months of age (Figure 1B). CRTAP expression, on the other hand, remained approximately the same from 3 weeks through 6 months of age (Figure 1C).

### 3.2. Tissue localization of SC65 and CRTAP expression in testis

**Figure 2.**
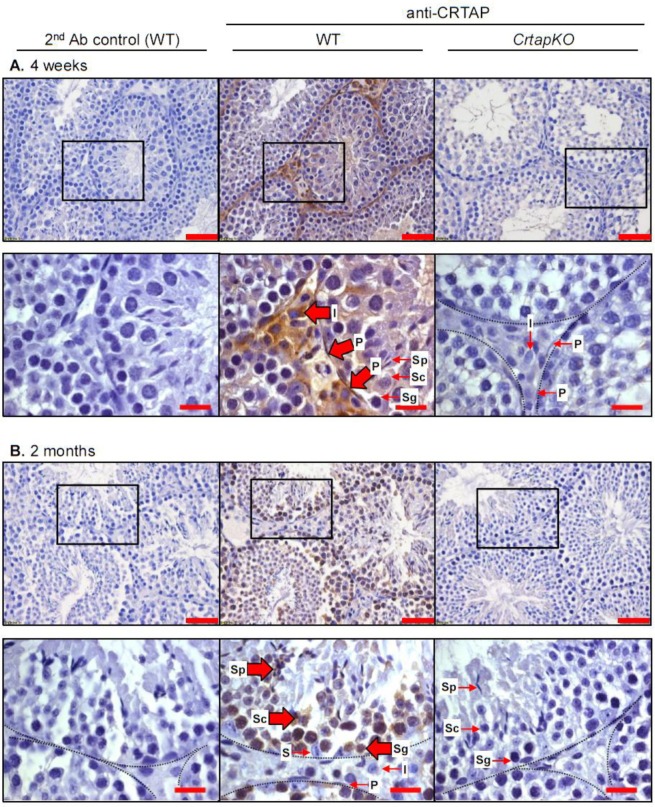
CRTAP is expressed in interstitial and peritubular cells in 4 week-old mouse testis, and in germ lineage cells in 2 month-old testis. IHC for CRTAP on testis from WT and *CrtapKO* mice at 4 weeks old (A) and 2 months old (B). The WT and KO tissues stained for CRTAP are the middle and right panels, respectively, and the control with secondary antibody only is to the left. The lower panels are higher magnification images corresponding to the areas indicated by the black boxes. Large arrows indicate cells with positive signal, and small arrows indicate negative cells. Where the morphology is unclear, dashed lines are provided to mark the boundaries of the seminiferous tubules. S: Sertoli cells, Sg: spermatogonia, Sc: spermatocytes, Sp: spermatids, P: peritubular cells, I: interstitial/stromal cells. Scale bars are 50 µm, and 20 µm in the higher magnification images.

Next, we wanted to determine which cell types in the testis express SC65 and CRTAP. Because we know that Leprecan proteins can participate in type I collagen modification, we expected to find SC65 and CRTAP in cells that contribute to ECM production, i.e. not in germ lineage cells. By immunohistochemistry (IHC), we detected SC65 in the peritubular cells (myoid cells) in 4 week-old testis (Table 2 and Figure S1). At 2 and 6 months of age, SC65 was no longer detectable in peritubular cells, however non-specific staining was associated with developing spermatids and other cells; similar staining was observed in the *Sc65KO* control tissue (Table 2 and Figure S1). This result is in agreement with the western blot data where SC65 was barely detectable after 4 weeks of age in testis (Figure 1B). CRTAP showed strong expression in interstitial tissue and peritubular cells in 4 week old mouse testis and, interestingly, weak expression was detected within the seminiferous tubules (Figure 2A). Beginning at 2 months of age, CRTAP expression was no longer apparent in the peritubular cells but was present in cells throughout all stages of spermatogenesis, indicating that CRTAP is expressed in both pre- and post-meiotic cells (Figure 2B).

**Table 2.** Summary of SC65 expression in testis assessed by immunostaining.

SC65 in testis		
Cell Type	4 weeks old	6 months old
spermatogonia	negative	background
spermatocytes	negative	background
spermatids	negative	background
Sertoli cells	negative	negative
peritubular myoid cells	**positive**	negative
interstitial cells	negative	background

**Figure 3.**
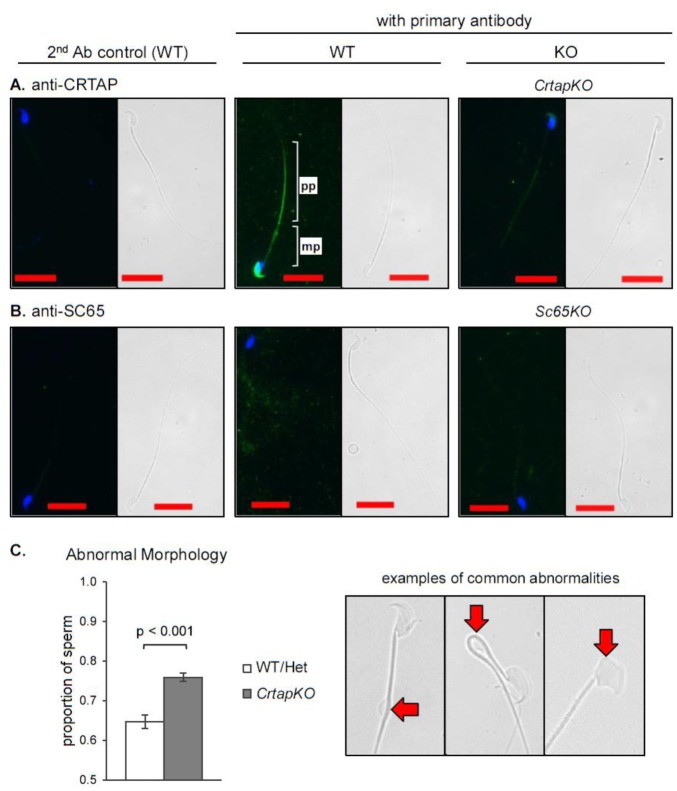
CRTAP but not SC65 is expressed in mature sperm in mice. Immunofluorescence for CRTAP (A) and SC65 (B) in mouse mature sperm. WT and KO sperm are in the middle and right panels, respectively, and secondary antibody controls are to the left. Nuclei are stained with DAPI, bright-field images are provided for reference, and the scale bars are 20 µm. pp: principle piece, mp: midpiece. C. Proportion of sperm with abnormal morphology observed in WT/Het and *CrtapKO* mature sperm from 5 month-old male littermates (n = 3, total sperm counted > 600/group). At right, representative images of the types of abnormalities observed.

### 3.3. CRTAP is expressed in mature sperm and its loss is associated with increased sperm morphological abnormalities

Because CRTAP is expressed in testicular germ cells, we examined its expression in mature sperm isolated from the caudal epididymis. CRTAP was detected by immunofluorescence (IF) to the principal piece of the sperm tail and the head (Figure 3A). This is a completely new finding and was rather unexpected, given CRTAP's known role in fibrillar collagen modification. We thus wondered if loss of CRTAP might somehow impact fertility. To investigate this further and in light of the potential expression of CRTAP by Leydig cells, we determined the levels of luteinizing hormone (LH), follicle stimulating hormone (FSH) and testosterone in serum of *CrtapKO* male mice but found no significant differences compared to WT (n = 6 of each genotype, Figure S2). No SC65 expression was detected in mature mouse sperm (Figure 3B). To assess whether loss of CRTAP has an impact on sperm morphology, mature sperm was collected from the caudal epididymides from 5 month-old mice (N = 3), and the morphology was analyzed by three independent, blinded researchers. The results showed a statistically significant 17% increase in sperm morphological alterations in *CrtapKO* compared to WT or heterozygous sperm (0.647 WT/Het, 0.759 *CrtapKO*, t_(df = 4)_ = 10.057, p = 0.00055, Figure 3C). The observed abnormalities included large cytoplasmic droplets, bent tails, and misshapen heads, and all types of abnormalities appeared in both groups (Figure 3C, right). Thus CRTAP is expressed in germ line cells in adult testis and in mature sperm, and its loss appears to mildly but significantly affect mature sperm morphology.

**Figure 4.**
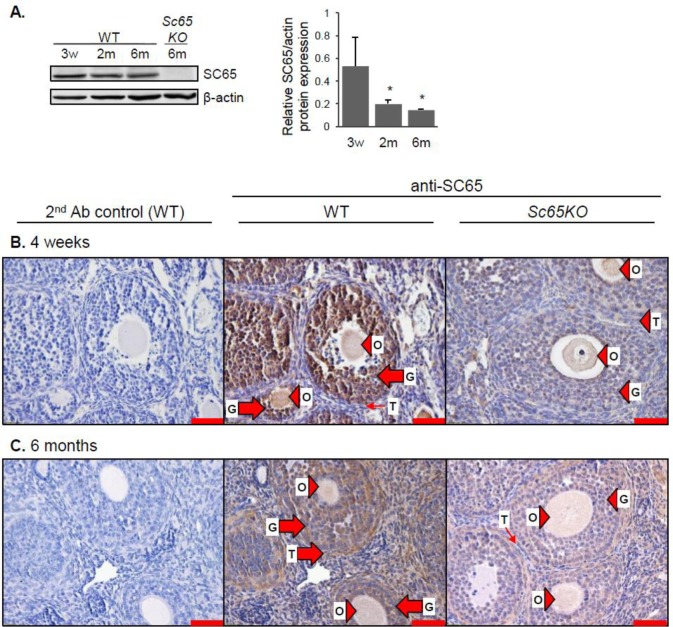
SC65 is expressed in follicular granulosa cells at 4 weeks and in granulosa, theca and stromal cells in 6 month-old mouse ovary. A. Western blot analysis for SC65 in WT and *Sc65KO* mouse ovaries at the ages indicated. Quantification by densitometry is shown to the right, normalized to beta-actin, n = 3 replicates, * p < 0.05 compared to 3 weeks old. B. and C. IHC for SC65 on ovary from WT and *Sc65KO* mice at 4 weeks old (B) and 6 months old (C). Large arrows indicate positive cells, small arrows indicate negative cells, and arrowheads indicate non-specific staining. O: oocyte, G: granulosa cells, T: theca cells. Scale bars are 50 µm.

### 3.4. SC65 and CRTAP expression and tissue localization in mouse ovary

**Figure 5.**
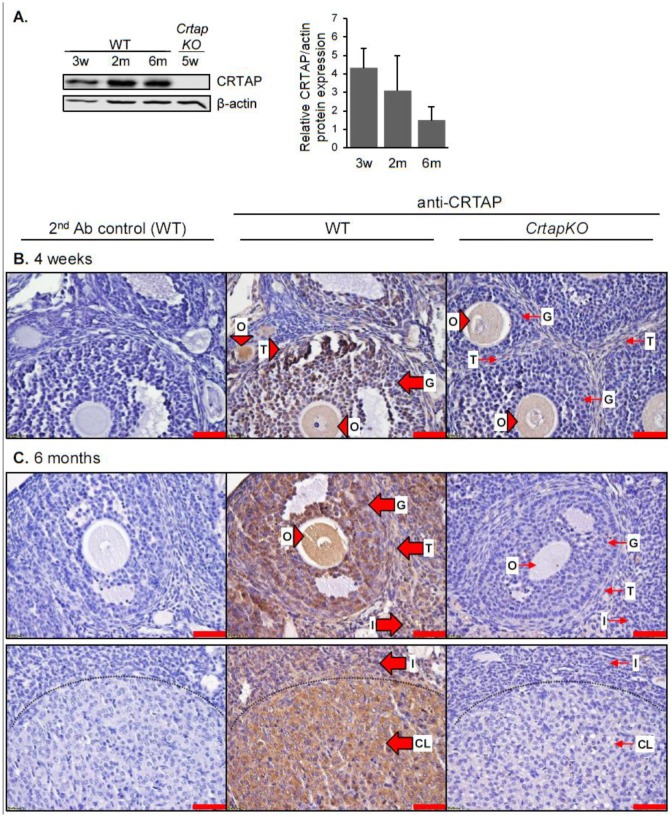
CRTAP is expressed in the follicular granulosa, theca cells and stromal tissue in mouse ovary. A. Western blot analysis for CRTAP in WT and *CrtapKO* mouse ovaries at the ages indicated. Quantification by densitometry is shown to the right, normalized to beta-actin, n = 3 replicates. B. and C. IHC for CRTAP in WT and *CrtapKO* mouse ovaries at 4 weeks of age (B) and 6 months of age (C). Large arrows indicate positive cells, small arrows indicate negative cells, and arrowheads indicate non-specific staining. The edge of the corpus luteum is indicated with a dashed line. O: oocyte, G: granulosa cells, T: theca cells, I: interstitial/stromal cells, CL: corpus luteum. Scale bars are 50 µm.

Given that both SC65 and CRTAP are expressed in testis, we also expanded our expression studies to the ovary. Western blot analysis showed that SC65 protein was present in the WT ovary of all ages but was more abundant in young versus adult mice (Figure 4A). In the 4 week-old ovary SC65 was abundantly expressed in the granulosa cells within preantral to provulatory-stage follicles, with little to no expression in the theca cells surrounding the follicles (Figure 4B). In the adult ovary SC65 was evident in the granulosa and theca cells, and additionally there was diffuse staining throughout the interstitial stromal tissue (Figure 4C). Only background staining was detected in the *Sc65KO* ovary (Figure 4B,C). For CRTAP, western blot analysis showed expression in the WT mouse ovary at all ages (Figure 5A). Staining for CRTAP was located in the granulosa and theca cells at every stage of follicular differentiation in the 4 week-old ovary, with less intense staining in the stromal tissue (Figure 5B). Conversely, the adult ovary showed CRTAP staining more equally distributed between the interstitial stromal tissue and the granulosa and theca cells of developing follicles (Figure 5C). Cells within a corpus luteum also expressed CRTAP (Figure 5C). No positive staining was observed in the *CrtapKO* ovary (Figure 5B,C).

### 3.5. Co-expression of SC65 and P3H3 in mouse testis and ovary

**Figure 6.**
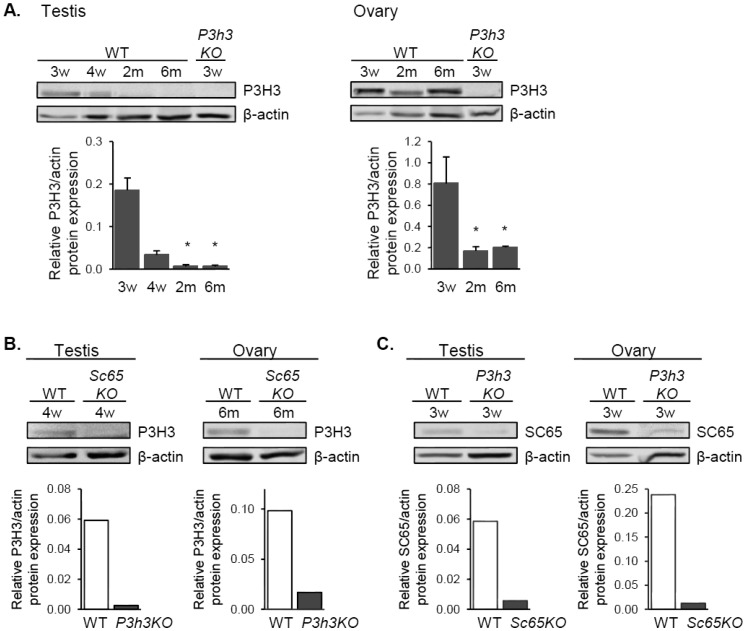
Co-expression of SC65 and P3H3 in mouse testis and ovary. A. Western blot analysis for P3H3 in testis (left) and ovary (right) of WT and *P3h3KO* mice at the ages indicated. Quantification by densitometry is shown below each blot, normalized to beta-actin, n = 3 replicates, * p < 0.05 compared to 3 weeks old. B. Western blot analysis for P3H3 in WT and *Sc65KO* gonads shows a dramatic decrease in P3H3 protein in *Sc65KO*, and similarly in C, SC65 protein decreases in *P3h3KO* gonads.

We have recently shown that SC65 and P3H3 are mutually co-stabilizing proteins within an ER complex in both osteoblasts and skin fibroblasts [13],[14]. Given their similar mRNA expression trend observed in testis (Figure 1A), we investigated if SC65 and P3H3 were also tightly co-expressed in gonads. Interestingly, western blot analysis for P3H3 on mouse testis and ovary showed protein expression similar to that of SC65, with detectable expression until 4 weeks of age in testis and higher expression in the ovary until adulthood (Figure 6A). Moreover, we found a dramatic loss of P3H3 protein in *Sc65KO* testis and ovary, and also a loss of SC65 protein in *P3h3KO* testis and ovary (Figure 6B,C respectively; the *P3h3KO* tissues were a kind gift of Dr. Brendan Lee) [14]. This relationship suggests that SC65 and P3H3 participate in the same ER protein complex in testis and ovary. Similarly, we found that P3H1 was expressed in mouse testis and ovary and was greatly decreased in *CrtapKO* tissues as previously described in bone and skin cells (Figure S3) [15],[16].

## 4. Discussion

In this study we explored the expression pattern of Leprecan proteins in mouse gonads since SC65 was initially described as a protein associating with the synaptonemal complex in rat testis [6] and currently the expression and/or role of Leprecans in reproductive organs is unknown. To do this we performed absolute quantification of Leprecan mRNAs from testis at different postnatal time points. These data show that all Leprecans are expressed in testis, and their expression tends to decrease with age. *P3h1* and *Crtap*, two components of the collagen prolyl 3-hydroxylation complex in connective tissues, are the most highly expressed with levels of expression that were comparable or higher to those observed in 1 month-old cortical bone. *P3h3* and *Sc65*, two of the components of the collagen lysyl-hydroxylation complex, are expressed at much lower levels and, based on western blot data, their expression is undetectable after 4 weeks of age in testis. *P3h2* transcripts are expressed at low levels and do not vary much with age. In addition, both the mRNA data and the protein data from testis and ovary confirm previous findings in osteoblasts and skin fibroblasts, i.e. the tight co-expression of CRTAP and P3H1 and of SC65 and P3H3 [13]–[16]. This suggests that CRTAP/P3H1 and SC65/P3H3 likely participate in the formation of the same collagen-modifying complexes in the ER of collagen-secreting cells in murine reproductive organs.

We find that SC65 is expressed in peritubular cells of the testis up until 4 weeks of age but not in the germ cells inside the seminiferous tubules or mature sperm. Peritubular myoid cells are not well studied, but are known to contribute to ECM formation in cooperation with Sertoli cells, and a normal ECM is essential for testicular function [17]. The observation that SC65 and P3H3 expression decreases in adult compared to 4 week-old testis and ovary indicates that they are post-natally regulated. The strong down-regulation of SC65 and P3H3 over puberty in testis could be explained by a decreased requirement for ECM remodeling in adult testes. In the ovary, SC65 is expressed at all tested ages including in granulosa cells of preantral to provulatory-stage follicles and the interstitial stroma but also in theca cells after 4 weeks of age. It is unclear whether oocytes express SC65 since the antibody for this protein yields some background, which also involves the oocyte in *Sc65KO* mice. The ovary expression of SC65 is more constant than the testis and may reflect the continual requirement for ECM remodeling that occurs as follicles and corpora lutea grow and regress [18].

In contrast to previous literature, our data did not support a role for SC65 in the SC because SC65 is not expressed in germ cells and it is barely detectable in testis after 4 weeks of age. However, it is also possible that SC65 expression levels in the SC could be too low to detect by IHC or western blot. If SC65 does associate with the SC, it is most likely not an essential SC component because we have observed that *Sc65KO* mice are fertile while most mice with knockouts of essential SC components suffer infertility [7],[19]–[25]. In addition, the results of a yeast 2-hybrid screen that we performed to identify SC65 candidate protein interactors did not include any proteins known to be associated with the SC, but functional annotation analyses pointed to spermatogenesis processes (Figure S4). Because SC65 and CRTAP are 55% identical at the amino acid level and share tetratricopeptide-like repeats (which are protein-protein interaction motifs), it is possible that some of the potential candidate interactors identified for SC65 may also interact with CRTAP.

CRTAP is expressed in stromal, follicular, theca cells and corpus luteum cells in the ovary although its expression in the oocyte is unclear due to some background staining in this cell. Its role in the ovary is likely involved in collagen production as part of the continual tissue remodeling of this organ as discussed above. The expression of CRTAP in peritubular and interstitial cells in the 4 week-old testis also suggests CRTAP may be involved in ECM production at this stage of development. At 4 weeks of age CRTAP expression was weak inside seminiferous tubules, but at 2 months of age was detected in germ cells at all stages of spermatogenesis in the adult testis. These unexpected findings led us to also identify expression of CRTAP in mature sperm. Whether CRTAP function in sperm is related to type I collagen is unknown, and there is controversial evidence about collagen expression inside the seminiferous tubules [26]. However, the prolyl-3-hydroxylation complex of which CRTAP is a part could have substrates other than collagen or could perform unknown functions in male germ cells. Clearly, CRTAP's function in mature sperm needs to be further studied, but its localization may suggest some intriguing possibilities. We observe CRTAP both in the head and in the principal piece of the sperm tail, suggesting localization to the acrosome and fibrous sheath and possible functions contributing to the maintenance or function of these structures.

*CrtapKO* male mice are fertile and have no significant changes in hormonal levels of LH, FSH and testosterone, although we cannot rule out a subfertility phenotype due to the increased sperm morphology alterations, which we have not tested. In our experience, *CrtapKO* males tend to produce smaller litters than WT males; however, the severe *CrtapKO* skeletal phenotype can affect mobility and thus the knockout male's ability to mate, and can cause in-utero or perinatal lethality resulting in a decreased litter count. Whether men with a diagnosis of OI due to biallelic mutations in *CRTAP* have decreased fertility remains an open question. *CrtapKO* mice had a small but significant 17% increase in morphologically abnormal sperm. While sperm morphology has long been used as a measure of damage to germ cells [27], and some types of morphological defects correlate negatively to *in vitro* fertilization ability [28], it is unclear how much of an increase in morphological defects would be necessary to affect a change in fertility in mice. Our measurement of approximately 65% abnormal sperm in WT or Crtap heterozygous mice appears high but it is comparable to results from a recent study [28].

## 5. Conclusion

Collectively, our data identified expression of Leprecan proteins in both murine testis and ovary. Our findings suggest a likely, conserved role of Leprecans in the post-translational modification of collagens expressed in the stromal tissue of the reproductive organs. However, the novel finding of CRTAP expression in the male germ line, including mature sperm, also points to a potential role for CRTAP in the spermatogenesis process that could be unrelated to the collagen synthetic pathway.
