# Supplementary material for: Expression characterization and functional implication of the collagen-modifying Leprecan proteins in mouse gonadal tissue and mature sperm
Source: AIMS Genet. 2018 Feb 7;5(1):24–40. doi: 10.3934/genet.2018.1.24 (PMC6221197; doi:10.3934/genet.2018.1.24)
Supplement: Supplementary file 1 [file genetics-05-01-024-s01.pdf]

AIMS Genetics

AIMS Genetics, 5(1): 24–40.
DOI: 10.3934/genet.2018.1.24
Received: 05 October 2017
Accepted: 30 January 2018
Published: 07 February 2018

http://www.aimspress.com/journal/Genetics

Research article

Expression characterization and functional implication of the collagen-modifying Leprecan proteins in mouse gonadal tissue and mature sperm

Sarah M. Zimmerman[1], Roberta Besio[1,#a], Melissa E. Heard-Lipsmeyer[1], Milena Dimori[1], Patrizio Castagnola[2], Frances L. Swain[3], Dana Gaddy[1,#b], Alan B. Diekman[4] and Roy Morello[1,3,5,*]

[1] Department of Physiology & Biophysics, University of Arkansas for Medical Sciences, Little Rock, AR, USA
[2] IRCSS AOU-San Martino-IST, Genoa, Italy
[3] Department of Orthopaedic Surgery, Center for Orthopaedic Research, University of Arkansas for Medical Sciences, Little Rock, AR, USA
[4] Department of Biochemistry, University of Arkansas for Medical Sciences, Little Rock, AR, USA
[5] Division of Genetics, University of Arkansas for Medical Sciences, Little Rock, AR, USA
[#a] Current Address: Department of Molecular Medicine, Universita' di Pavia, Pavia, Italy
[#b] Current Address: Department of Veterinary Integrative Biosciences, College of Veterinary Medicine and Biomedical Sciences, Texas A&M University, College Station, TX, USA

* **Correspondence:** Email: rmorello@uams.edu; Tel: +5015264090, +5015264091.

Supplementary

S1 Text. Materials & methods for supporting figures.

Testing of hormone serum levels

For hormone testing, n = 6 male WT and *CrtapKO* mice were individually housed for a minimum of 2 weeks prior to serum collection, which was performed when the mice were

approximately 10 weeks old. Serum samples were sent to the University of Virginia's Center for Research in Reproduction. The concentrations of luteinizing hormone (LH), follicle stimulating hormone (FSH), and testosterone in the serum were quantified via radioimmunoassay. The University of Virginia Center for Research in Reproduction Ligand Assay and Analysis Core is supported by the Eunice Kennedy Shriver NICHD/NIH (NCTRI) Grant P50-HD28934.

Yeast 2-hybrid screen for protein interactions

The Matchmaker Gold Yeast Two-Hybrid System from Clontech was used. A cDNA construct expressing full-length murine Sc65 was subcloned into pGBKT7 vector as EcoR1/Sal1 insert, verified by sequencing, and transformed into yeast AH109 mate A using the Frozen-EZ Yeast Transformation II kit (T2001 Zimo Research). Expression of the Sc65 hybrid molecule was verified by Western blot. The universal mouse cDNA library coding for 2.8x106 proteins (cat.# 630482 Clontech) was transformed into yeast strain Y187 as prey. The mating of AH109 Mate A pGBKT-53 with Y187 Mate a pGADT7-T was used as positive control, whereas the negative controls were the matings of AH109 Mate A pGBKT-Lam with Y187 Mate a pGADT7-T and of AH109 Mate A pGBKTSC65 with Y187 Mate a pGADT7-empty. Growth of transformants was assessed on medium lacking leucine (-LEU, prey fusions) or tryptophan and uracil (-TRP/URA, bait fusions). Matings were performed at 30 °C at 50 rpm overnight in liquid -LEU/TRP medium. The mating efficiency was 20%. Diploids were grown in liquid -LEU/TRP medium to an optical density at 600 nm of 0.8 and titrated 1:10, 1:100, 1:1,000, and 1:10,000 in ddH2O. Each dilution was applied on a selective agar plate (-ADE/HIS/LEU/TRP), incubated at 30 °C for 72 h, and scored for growth. Diploid yeasts were assessed for the stringency of direct protein-protein interaction by growth assay on -HIS agar plates (low), and -ADE plates (high) and plates containing 5 mM 3-amino-1,2,4-triazole (Sigma A8056). All positive colonies were replicated on a YPD -LEU/URA/TRP/ADE plate containing x-gal and 5 mM 3-amino-1,2,4-triazole. The 1770 blue colonies were screened by PCR, amplicons digested by AluI to reduce clone redundancy, and the resulting 768 non-redundant clones were sequenced (Beckman Coulter Genomics). The sequences were identified using BLAST, and the number of sequences aligning to a gene (number of "hits") were totaled and used to generate a list of candidate interactors (Figure S4) Functional annotation enrichment analysis was performed on the list of yeast 2 hybrid results using DAVID software (Figure S4).

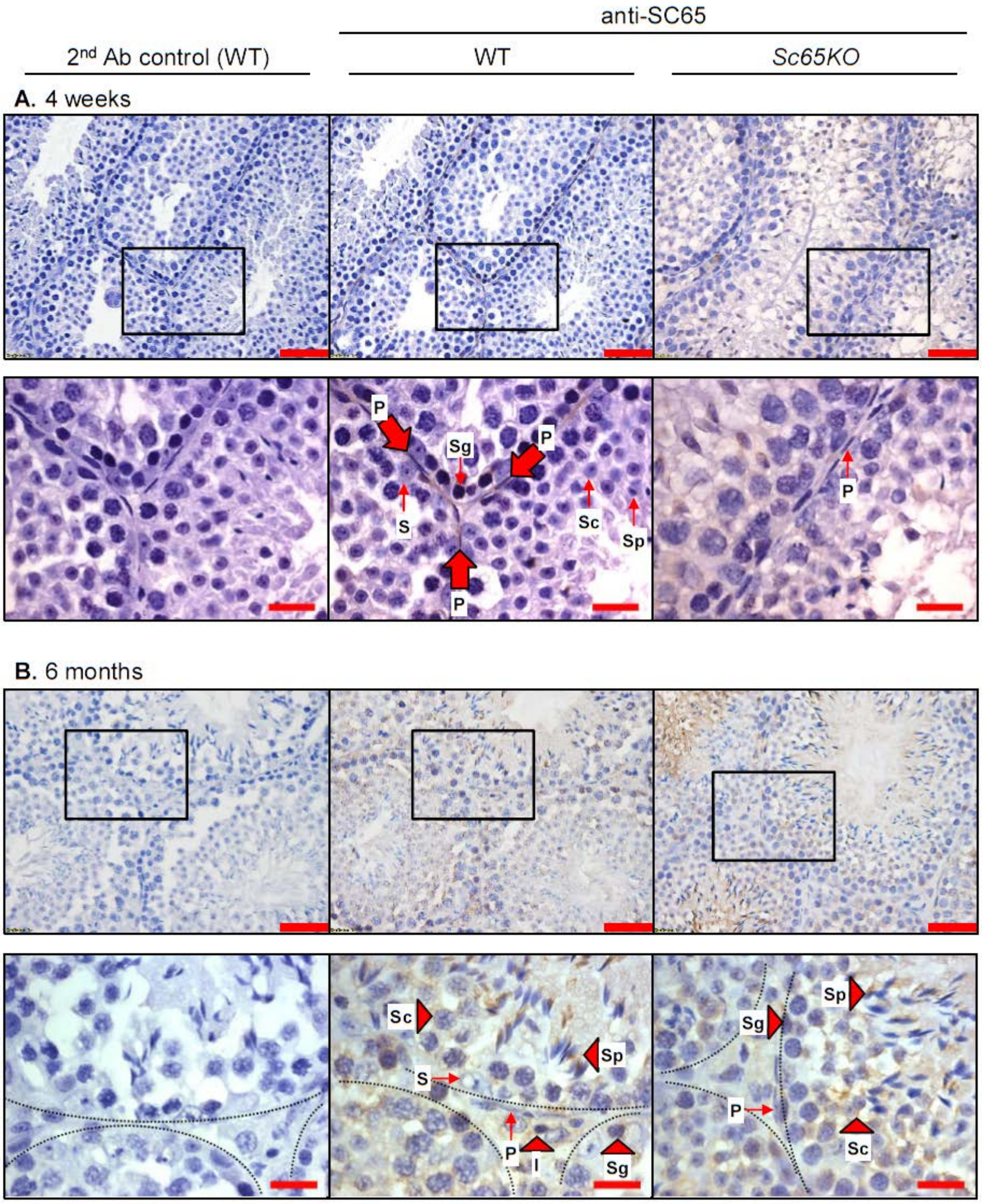

Figure S1. SC65 is expressed in peritubular cells in 4 week-old mouse testis. IHC for SC65 on testis from WT and *Sc65KO* mice at 4 weeks (A) and 6 months of age (B). The WT and KO tissues stained for SC65 are shown in the middle and right panels, respectively, and the control with secondary antibody only is to the left. The lower panels are higher magnification images corresponding to the areas indicated by the black boxes. Large arrows indicate cells with positive signal, small arrows indicate negative cells, and arrowheads indicate non-specific staining. Where the morphology is unclear, dashed lines are provided to mark the boundaries of the seminiferous tubules. S: Sertoli cells, Sg: spermatogonia, Sc: spermatocytes, Sp: spermatids, P: peritubular cells, I: interstitial/stromal cells. Scale bars are 50 µm, and 20 µm in the higher magnification images.

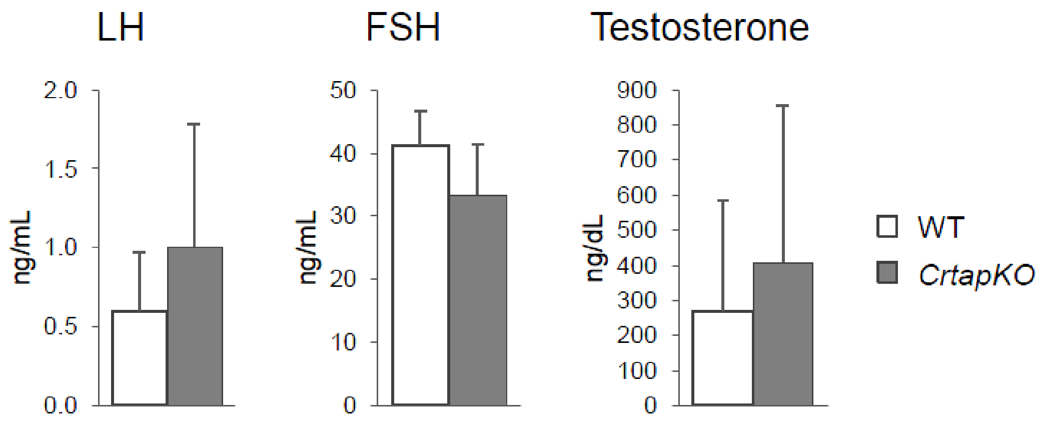

Figure S2. Hormone serum levels are unchanged in *CrtapKO* mice. No changes were detected in the serum concentrations of luteinizing hormone (LH), follicle stimulating hormone (FSH) and testosterone in 10 week-old *CrtapKO* vs WT male mice (n = 6).

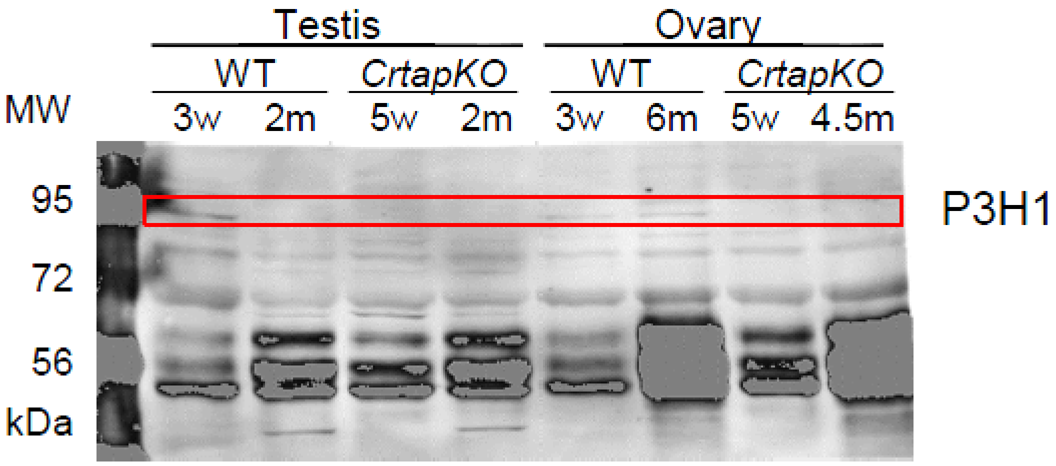

Figure S3. P3H1 was detected in WT mouse gonads but was absent in *CrtapKO* mice. Western blot analysis detected a faint band for P3H1 in the testis and ovary of young and adult WT mice that was not present in *CrtapKO* mice (ages are indicated, w = week, m = month). The red box indicates the P3H1 band.

A. Yeast 2-Hybrid Results

	Protein Name (Gene Symbol)	Hits
1	spermatogenesis-associated protein 4 (*Spata4*)	158
2	spermatogenesis-associated protein 3 isoform 2, 3 (*Spata3*)	60
3	centrosomal protein of 57 kDa (*Cep57*)	36
4	coiled-coil domain containing protein 181 (*Ccdc181*)	36
5	cilia- and flagella-associated protein 97 (*Cfap97*)	20
6	N-myc downstream regulated gene 2 protein isoform 1, 2 (*Ndrg2*)	17
7	C1D nuclear receptor co-repressor (*C1d*)	16
8	small VCP/p97-interacting protein (*Svip*)	13
9	kinesin family member 3A isoform 1, 3 (*Kif3a*)	12
10	nuclear fragile X mental retardation interacting protein 1 (*Nufip1*)	10
11	spermatogenesis-associated protein 1 (*Spata1*)	9
12	G-protein signaling modulator 2 (*Gpsm2*)	9
13	male specific lethal 1 homolog (*Msl1*)	8
14	TBC1 domain family member 15 (*Tbc1d15*)	8
15	uncharacterized protein C8orf48 homolog (*AI429214*)	7

B. Functional annotation (GO term) enrichment analysis with DAVID software

GO Term	p-value
gamete generation	0.0081
spermatogenesis	0.011
male gamete generation	0.011
sexual reproduction	0.016
reproductive process in a multicellular organism	0.021
multicellular organism reproduction	0.021
negative regulation of macromolecule biosynthetic process	0.023
negative regulation of cellular biosynthetic process	0.026
negative regulation of biosynthetic process	0.027
spermatid development	0.039
mitotic cell cycle	0.040
spermatid differentiation	0.044
regulation of blood pressure	0.046

Figure S4. Results of a yeast 2-hybrid screen to find SC65 interactors and the functional annotations enriched in this list of candidate interactors. A. The top 15 candidate interactors of SC65 from the yeast 2-hybrid screen. The list is ordered by the number of sequenced clones that matched to that particular gene ("Hits"). B. DAVID software was used to identify functional annotations or GO terms which were enriched in the list of candidate interactors. This table shows all of the GO terms in the biological process category with a p-value < 0.05.

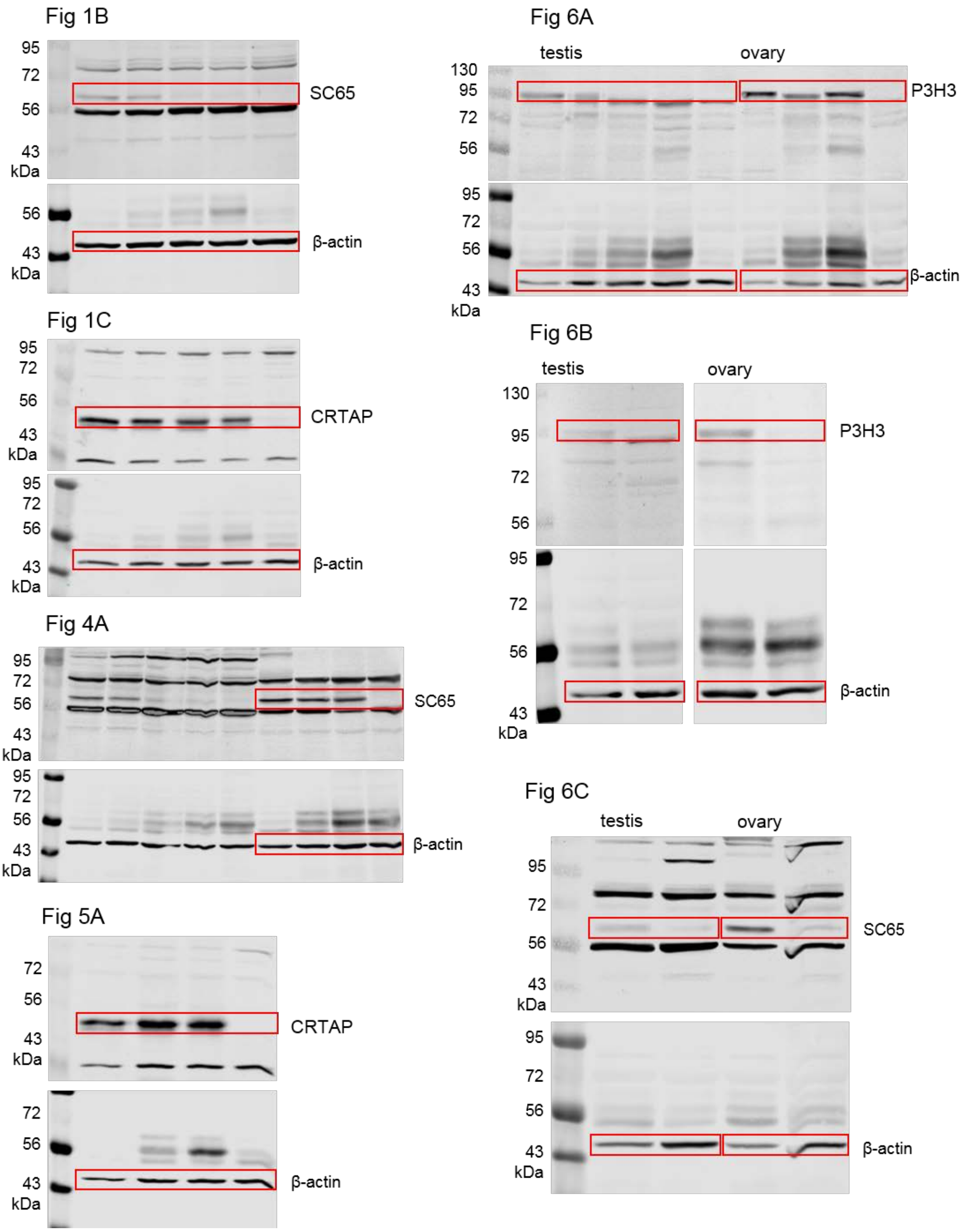

Figure S5. Uncropped immunoblotting images. Uncropped images of immunoblots corresponding to Figures 1B, 1C, 4A, 5A, 6A, 6B and 6C.

Table S1. Information on the primary antibodies used in this study.

Antibody	Target	Mono- or Poly-clonal	Host	Supplier	Catalog#	RRID/ Reference
Sc65 Polyclonal Antibody	Sc65	Poly	Rabbit	Proteintech Group	15288-1-AP	AB_2184611
Crtap Antibody	Crtap	Poly	Rabbit	N/A	N/A	Ref.#1
LEPRE1 Antibody (3C7)	P3h1 (Lepre1, Leprecan)	Mono	Mouse	Novus	H00064175-M01	AB_539369
Beta-Actin (8H10D10) Mouse mAb	β-actin	Mono	Mouse	Cell Signaling Technology	3700	AB_2242334
LEPREL2 Polyclonal Antibody	P3h3 (Leprecan-like 2)	Poly	Rabbit	Proteintech Group	16023-1-AP	AB_2281291

AIMS Press

© 2018 the Author(s), licensee AIMS Press. This is an open access article distributed under the terms of the Creative Commons Attribution License (http://creativecommons.org/licenses/by/4.0)
